# Carrageenans as Broad-Spectrum Microbicides: Current Status and Challenges

**DOI:** 10.3390/md18090435

**Published:** 2020-08-21

**Authors:** Choongho Lee

**Affiliations:** College of Pharmacy, Dongguk University, Goyang 10326, Korea; lkj640@gmail.com; Tel.: +82-31-961-5223

**Keywords:** sulfated polysaccharides, carrageenans (CGs), broad-spectrum microbicides, in vitro and in vivo toxicity, in vitro, ex vivo, in vivo antiviral activity

## Abstract

Different kinds of red algae are enriched with chemically diverse carbohydrates. In particular, a group of sulfated polysaccharides, which were isolated from the cell walls of red algae, gained a large amount of attention due to their broad-spectrum antimicrobial activities. Within that group, carrageenans (CGs) were expected to be the first clinically applicable microbicides that could prevent various viral infections due to their superior antiviral potency and desirable safety profiles in subclinical studies. However, their anticipated beneficial effects could not be validated in human studies. To assess the value of a second attempt at pharmacologically developing CGs as a new class of preventive microbicides, all preclinical and clinical development processes of CG-based microbicides need to be thoroughly re-evaluated. In this review, the in vitro toxicities; in vivo safety profiles; and in vitro, ex vivo, and in vivo antiviral activities of CGs are summarized according to the study volume of their target viruses, which include human immunodeficiency virus, herpesviruses, respiratory viruses, human papillomavirus, dengue virus, and other viruses along with a description of their antiviral modes of action and development of antiviral resistance. This evaluation of the strengths and weaknesses of CGs will help provide future research directions that may lead to the successful development of CG-based antimicrobial prophylactics.

## 1. Introduction

Microbicides are disinfectants designed to eliminate the infectivity of pathogenic microorganisms. In general, they render infectious microbes non-viable and prevent further transmission. When they are directed against viruses, they are often called virucides. The main antiviral mechanism of virucides involves chemical and physical destruction of the virus particle’s structural components (i.e., envelope, enveloped glycoprotein, capsid, and nucleic acid). Unlike conventional antiviral drugs that specifically target essential viral and host functions necessary to complete the virus life cycle, virucides exert their antiviral actions primarily by targeting the viral entry step via direct disruption of the actual virus particles in a relatively less specific manner. For this reason, the systemic internal administration of microbicides is not recommended due to safety concerns. Instead, the external application of microbicides is the preferred administration route for the prevention of microbial transmission.

**Figure 1:**
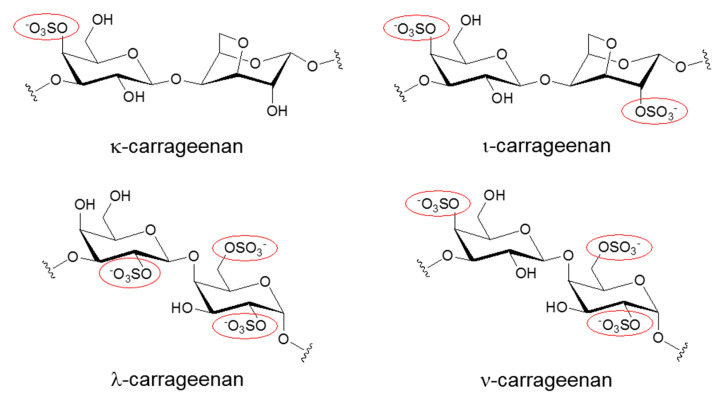
Chemical structures of different Carrageenans (CGs) including kappa (κ), iota (ι), lambda (λ), and nu (ν) forms. Only the repeating disaccharide unit of each CG is shown here. To emphasize the degree of sulfation, sulfated groups are highlighted with red circles.

Carrageenans (CGs) are negatively charged polysaccharides isolated from the cell walls of red algae [1,2,3]. Along with cellulose sulfate and naphthalene sulfonate, CGs belong to a group of sulfated polysaccharides with a broad antiviral spectrum [4]. In particular, kappa (κ) and iota (ι) forms of CGs are linear polymers with a repeating disaccharide unit composed of sulfated galactose and anhydrogalactose, whereas the composition of lambda (λ) form of CG is solely based on sulfated galactose without 3,6-anhydrogalactose (Figure 1). These two basic carbohydrate building blocks are connected via α-1,3 and β-1,4 glycosidic linkages. Native carrageenan always present complex hybrid structures are generally a mixture of galactans composed of different carrabiose types, proportions, and structure of which vary with species algae. The number of sulfate groups dictates the physical property of these sulfated polyanionic substances. For example, κ-CG, containing one sulfate, forms a rigid gel through a helix formation in ionic solutions, but iota ι-CG, containing two sulfates, forms a flexible gel. In line with this trend, lambda λ-CG, containing three sulfates, does not possess a gel-forming property because it is devoid of 3,6-anhydrogalactose [5]. λ-CG is more frequently used as a thickener or a stabilizer instead. Due to their gelling, thickening, and stabilizing characteristics, CGs are widely used in the food industry. Recently, these sulfated polysaccharides showed promise as potential microbicides in the prevention of a variety of viral diseases [1,2,3,4,6]. CGs have demonstrated efficient neutralization of several clinically relevant viruses including human immunodeficiency virus (HIV), herpes simplex virus (HSV), influenza virus, human papillomaviruses (HPV), and dengue virus (DENV). In particular, the topical vaginal application of CGs protected mice against several sexually transmitted infections (STI) induced by both HIV and HSV [7,8,9]. Therefore, they were proposed as the most suitable candidates in a multipurpose STI prevention strategy that involves blocking the transmission of several STI-related viral infections at once. Also, the development of CG-based microbicides as pre-exposure prophylaxis in the form of topical gels was expected to provide a self-protective tool for women [10]. This may further contribute to the reduction of gender inequality arising from male-dominant decisions on sexual behavior. However, despite all of these promising results from subclinical in vitro and in vivo studies, clinical trials of several sulfated polysaccharides showed no beneficial effects as preventive measures against major viral infections [11,12]. In some cases, sulfated polysaccharides, paradoxically, enhanced viral infections, and caused a major drawback in their clinical development [13,14,15].

To reassess the feasibility of the second round of CG pharmacological development to create a new class of preventive microbicides, all preclinical and clinical development processes of CG-based microbicides need to be thoroughly re-evaluated. Therefore, this review aims to provide a full picture of the current status and future challenges of developing CGs as broad-spectrum prophylactic microbicides. To this end, the in vitro toxicities and in vivo safety profiles of the most studied CGs are re-examined first. Then, their known common antiviral modes of action are explored at the molecular level. As HIV is the most studied antiviral target for CGs, their in vitro, ex vivo, and in vivo anti-HIV activities are re-examined in detail. In particular, the unexpected enhancement of HIV infectivity by CGs is also discussed. Concerning the anti-herpesvirus activities of CGs (herpesvirus is the second most studied antiviral target of CGs), the in vitro and in vivo effects of CGs on various types of herpesviruses, including HSV type 1, HSV type 2, and cytomegalovirus (CMV), are also described. In particular, the development of CG resistance by HSV is also discussed. Considering that the development of preventive measures against ongoing coronavirus-related pandemics is important, the effects of CGs on respiratory viruses such as rhinovirus, influenza virus, and coronavirus are also updated along with their effects on other viruses. Based on the strengths and weaknesses of CGs, a future research direction for a successful second round of CG development as a new class of preventive microbicides is proposed in the conclusion section.

### In Vitro Toxicity and In Vivo Safety

The half-maximal cytotoxicity concentration (CC_50_) is the gold standard when assessing the in vitro toxicity of potential antiviral candidates. Therefore, the CC_50_ values of CGs in different cell lines used in virus research were determined. The cell lines include MDCK cells, Vero cells, C6/36 HT mosquito cells, MDBK cells, BSR cells, MT-4 cells, PRK cells, HeLa cells, PLC/PRF/5, HepG2 cells, foreskin PH cells, mouse astrocytes, and BHK-21 cells. As shown in Table 1, their CC_50_ values range from 5 to 3000 µg/mL depending on the types of CGs and cell lines used [16,17,18,19,20,21,22,23,24,25,26,27,28,29,30,31,32,33,34,35,36,37,38,39,40,41,42]. Although more sulfated CGs such as λ-CG seems to be less toxic to cells than less sulfated CGs such as κ-and ι-CGs, it was difficult to draw any general relationship between the degree of sulfation and the cytotoxicity due to the presence of many outliers (Table 1). The unspecified CG used in the study by Huang et al. showed the lowest CC_50_, which was 5 µg/mL [29]. This might be due to their use of the unconventional cell viability quantitation method based on cellular oxygen consumption rates instead of a more traditional tetrazolium dye-based method [29]. The different origins of each cell line might also be responsible for the diverse effects of CGs on cell viability. Nevertheless, most in vitro CG studies demonstrated their superior cytotoxicity profiles; the highest CC_50_ values surpassed 1000 µg/mL, which seems to be an unattainable concentration in in vivo application. However, the wide range of CC_50_ values of CGs needs to be taken into account when CGs are applied to different tissues in vivo.

Table 2 shows the in vivo safety profiles of three types of CG gels including ι-CG (PC-213), κ/λ-CG (PC-515), and κ/λ-CG as well as a nonnucleoside reverse transcriptase inhibitor (MIV-150) and zinc acetate (ZA) (PC-1005) in eleven different clinical studies that recruited a variety of different cohorts based on HIV and sexual activity status. They were instructed to administer different types of CG gels with varying frequency and duration. Most of the clinical studies reported very high acceptability of CG gels because they did not irritate the female reproductive tract [44], caused no epithelial disruption [45], caused no vaginal floral change [46], and most importantly, did not increase proinflammatory cytokines in cervicovaginal lavages (CVLs) [47]. One study even reported increased sexual pleasure by using CG gel [48]. The addition of other pharmacological ingredients such as MIV-150 and ZA had no deleterious effects on the overall safety and acceptability of CG gels [49]. These data further indicate the superior safety profiles of CG gels when used alone or in conjunction with other preexisting anti-HIV therapeutics. Along with these desirable safety features, CGs also demonstrated effective anti-HIV efficacy. This was demonstrated by a lack of HIV RNA genital shedding [46], reduced HIV viral loads [50], and maintenance of anti-HIV activity in CVLs [49]. These desirable in vitro and in vivo safety properties demonstrated by CGs accelerated their clinical development.

## 2. Mechanisms of Antiviral Action

Before describing the antiviral effects of CGs on several clinically relevant viruses in detail, their common antiviral mode of action needs to be explained at the molecular level for a better understanding of their common pharmacological actions. In general, the electrostatic interactions between the anionic groups in the polysaccharide (mainly sulfates) and the basic amino acids of the virus glycoprotein is an essential requirement for the initial adsorption of a virus particle to the cell surface [2]. Particularly, the non-specific interaction of a virus glycoprotein with heparan sulfate on the cell surface serves as a necessary first step in the engagement of host receptors by a virus for successful viral entry. As a structural mimic to heparan sulfate, CGs form a complex with viral glycoproteins that prevents them from binding directly to the extracellular heparan sulfate. This blocks viral attachment and subsequent viral entry into a host cell.

In the case of HIV, CGs shield the positively charged sites of the HIV envelope glycoprotein (gp120) via electrostatic interactions [4]. Since this HIV-heparan sulfate interaction is necessary for initial HIV attachment to the cell surface before binding to the CD4 molecule of T cells, the interaction of CGs with gp120 interferes with the CD4-binding function of gp120, thereby blocking HIV entry [3]. In addition to this virus-targeting antiviral mechanism, CGs also exert antiviral actions via the inhibition of intercellular viral transmission [3]. For example, CGs inhibit the transfer of HIV from HIV-infected lymphocytes to uninfected epithelial cells [55]. The suppression of this intercellular HIV trafficking by CGs is thought to play a major role in the overall antiviral activity of CGs since the inhibitory concentrations required to target intercellular HIV trafficking by CGs were one thousand-fold lower than those required to target HIV entry [55].

Another host-targeting antiviral mechanism employed by CGs involves their direct binding to the CD4 protein of T cells [56]. Once bound to CGs, CD4 cannot interact with gp120, preventing infection of CD4-positive T cells by HIV [56]. In line with this, the association of CGs with CD4 inhibited anti-CD4 monoclonal antibodies from binding to CD4, itself [56]. However, the ability of gp120 to bind to monocytes was not affected by CGs, demonstrating their cell-type-specific antiviral effects [56].

Although the detailed molecular mechanism for these cell-type-specific antiviral activities of CGs is not clear, the difference in the proteoglycan compositions between T cells and monocytes may play a role in the differential antiviral actions of CGs. However, the antiviral activity of CGs tends to increase with either a higher molecular weight or a higher degree of sulfation [3,57,58]. Since antiviral activities of CGs mainly depend on neutralization of positively charged residues on either viral structural proteins or host receptors through electrostatic interaction, the increased negative charges on CGs by higher sulfation should contribute to the enhancement of the overall antiviral activities of CGs. In addition, the specific position of the sulfate ester group appears to be another critical determinant for the antiviral activity of sulfated polysaccharides [58]. Although the polyanionic nature of CGs is a critical factor for antiviral activity, the type of the anionic group also seems to be important. For example, carboxyl groups generally do not promote antiviral activity [58]. Thus, the antiviral activity is not merely a function of high charge density but has distinct structural specificities. Therefore, the nature of the negatively charged group and its position seem to influence their overall antiviral activities. The composition of the repeating carbohydrates of CGs affects their antiviral potency. Although one study demonstrated the higher antiviral potency of sulfated homopolysaccharides than sulfated heteropolysaccharides [57], this observation does not seem to be generalizable due to the presence of many other studies which suggests the opposite trend [58,59,60]. Of note, one group of researchers reported the selective inhibition of HIV reverse transcriptase and viral replication in vitro by a sea algal extract containing CGs; however, the exact intracellular anti-HIV mechanisms that are independent of virus entry disruption are not known [61]. Another group of researchers also reported a distinct antiviral mechanism of CGs that does not target virus entry [62,63]. They found that augmentation of natural killer cells and CG-induced infiltration of polymorphonuclear neutrophils inhibited the spread of murine cytomegalovirus from the peritoneal cavity to the plasma [62,63]. These two novel antiviral mechanisms exhibited by CGs deserve further attention to better understand CG virus-entry-independent antiviral properties. For those who wish to understand the in-depth antiviral mechanisms of action with a nice illustration, please refer to the following reference [6].

## 3. Human Immunodeficiency Virus (HIV)

### 3.1. In Vitro Anti-HIV Activity

HIV infection causes acquired immune deficiency syndrome (AIDS). Owing to the introduction of highly active antiretroviral therapy, AIDS became a clinically manageable disease. Nevertheless, continuous failures in developing HIV vaccines and a lack of effective preventive measures have been large hurdles in effectively controlling new HIV infections. Those who live in resource-poor and HIV-endemic regions have been most vulnerable to these recurring and uncontrolled HIV infections. Also, the unavailability of self-protective devices further aggravated the already uncontrollable state of new HIV infections [64]. Furthermore, the practice of unprotected receptive anal intercourse facilitated the spread of new HIV infections due to enriched HIV target cells in traumatized rectal mucosa [65]. Therefore, there is a desperate need for new HIV prevention strategies that mitigate HIV transmission. In this regard, CG-based topical anti-HIV gels were proposed as a promising on-demand, pre-exposure prophylactic option for the public. The antiviral activities of CGs in vitro, ex vivo, and in vivo along with the paradoxical increase of HIV infectivity by CGs are summarized below.

One of the most frequently used methods to assess the in vitro potency of potential antiviral candidates is the determination of their half-inhibitory concentration (IC_50_). For this purpose, the IC_50_ values of different CGs in several HIV-infected cell lines such as MT-4, CEM-SS, TMZ.bI, dendritic (DC), HeLa-CD4-CCR5, and P4-R5 MAGI cells were determined by using various anti-HIV assays (Table 3). These assays include plaque/cytopathic reduction assays, p24 ELISAs, luciferase assays, and microtiter syncytial assays. Their IC_50_ values ranged from 0.03 to 100 µg/mL depending on the cell type and experimental system. The IC_50_ values of most CGs were approximately 30- to 160-fold lower than their CC_50_ values, suggesting wide therapeutic windows, which is the most desirable feature for effective and safe pharmacological use (Table 1).

As mentioned previously, CGs such as ι-CG inhibit HIV entry into lymphocytes and lymphocyte-to-epithelial transmission of HIV [55]. Interestingly, CG-induced suppression of lymphocyte-to-epithelial transmission of HIV occurred at concentrations one thousand times lower than the IC_50_ concentrations necessary for inhibition of HIV entry [55]. Since viral transfer is a critical step in successfully establishing HIV infection in the target cells, this mechanistically distinct antiviral mode of action might be useful for achieving maximal inhibition of HIV infection by CGs. On the other hand, the polymerization status of CGs seems to be an important determinant for their antiviral action since the depolymerized κ- and ι-CGs demonstrated much higher anti-HIV activities than native polymerized CGs [66]. In addition, direct covalent modification of CGs with other types of anti-HIV therapeutics synergized their antiviral activities. For example, when κ-CG was covalently conjugated with 3′-azido-3′-deoxythymidine (AZT, a reverse transcriptase inhibitor), its anti-HIV activities were enhanced [41]. The combined treatment of λ-CG with MIV-150 also reduced the original IC_50_ value of λ-CG by approximately 10 times [67]. These data indicate the utility of chemically modifying CGs or using them in combination with other antiviral drugs to improve their overall antiviral potency. As mentioned previously, higher CG antiviral activity is generated by increased sulfation. This was evident since λ-CG exhibited reduced IC_50_ values when compared to those of κ- and ι-CGs (Table 3).

### 3.2. Ex Vivo Anti-HIV Activity

Five studies examined the anti-HIV activity of λ/κ-CG in conjunction with MIV-150 and ZA ex vivo by using human cervical and macaque vaginal explants (Table 4). In general, human cervical and macaque vaginal tissues are excellent ex vivo models for testing the antiviral activities of candidate compounds against HIV infection because of their high in vivo similarity. These ex vivo studies reported that λ/κ-CG, alone or in conjunction with MIV-150 and ZA, successfully suppressed HIV infection. In particular, residual λ/κ-CG/MIV-150, which was retrieved from the CVLs of human subjects after vaginal administration, maintained its inhibitory effects on HIV infections in human cervical explants [9]. In another study, vaginal application of MIV-150/ZA gel inhibited simian-human immunodeficiency virus reverse transcriptase (SHIV-RT) infection in macaque vaginal explants derived from monkeys when applied vaginally in a similar ex vivo setting [69]. Similarly, the addition of λ/κ-CG in combination with MIV-150/ZA showed much greater anti-SHIV-RT activity than tenofovir gel in macaque vaginal explants [7]. In addition, this triple combination (λ/κ-CG/MIV-150/ZA) gel completely protected macaque vaginal explants against free and cell-associated SHIV-RT infection [70]. These data further validate the superior antiviral activities of CGs in ex vivo conditions.

### 3.3. In Vivo Anti-HIV Activity

Before testing them in a human clinical trial, the anti-HIV activities of CGs were first tested in mouse and macaque animal models (Table 5). In one mouse model, a λ/κ-CG formulation blocked macrophage trafficking from the vagina to the lymph nodes [72]. Since the movement of macrophages from the vagina to lymph nodes is an essential step for HIV spread after initial infection, the specific blockage of this macrophage trafficking by CGs may delay the transmission of HIV to other parts of the body. When λ/κ-CG was applied with either MIV-150, alone, or MIV-150/ZA as either vaginal or rectal gels, it also successfully diminished vaginal and rectal transmission of SHIV-RT infection in both vaginally and rectally challenged macaque models (Table 5) [7,8,12,73,74]. Of note, the addition of MIV-150 did not increase the antiviral activity of λ/κ-CG in vivo. This is likely due to the limited activity of an MIV-150 single-dose and the dominant barrier effect of λ/κ-CG [12]. The λ/κ-CG/MIV-150/ZA triple combination also demonstrated antiviral efficacy against rectal infection when administered close to the time of SHIV-RT exposure [75]. Vaginal SHIV-RT infection was also attenuated when macaques were challenged after the administration of λ/κ-CG plus ZA gel with modified buffers and cosolvent [8]. However, λ/κ-CG, alone, showed limited activity against cell-free and mature DC-driven SHIV-RT infections [12]. Although low doses of λ/κ-CG enhanced SHIV-RT infection, the addition of MIV-150 with λ/κ-CG treatment overcame this enhancement effect and blocked DC-transmitted HIV infection [12]. Despite these positive and preventive effects of CGs in different in vivo animal models, a randomized, double-blind, and placebo-controlled clinical study using 6202 HIV-negative women found similar HIV incidence rates and time to seroconversion in both placebo and λ/κ-CG-treated groups [11]. Although the safety profiles of λ/κ-CG gel were acceptable in this human study, the further development of CGs as a new class of anti-HIV microbicides was halted when λ/κ-CG demonstrated no beneficial effects on the prevention of vaginal transmission of HIV [11].

### 3.4. Enhancement of HIV Infectivity

Two studies noticed a seemingly paradoxical enhancement of HIV infection in the presence of polyanionic compounds [12,68]. Similarly, cellulose sulfate, which is a structural analog of CGs, also increased the risk of HIV infection [76]. In particular, cellulose sulfate showed a dose-dependent biphasic effect on HIV infection in vitro [15]. In this study, low concentrations of cellulose sulfate significantly and reproducibly increased HIV infection in in vitro experiments [15]. Similarly, in in vitro “washout” experiments, λ-CG also significantly enhanced HIV infection despite potent antiviral activity at higher concentrations [13]. This enhancement of HIV infection by a low dose of cellulose sulfate was further supported by one clinical study [77]. In this study, a higher number of newly acquired HIV infections was observed in the cellulose sulfate-treated patient group than the placebo group [77]. The exact molecular mechanism for concentration-dependent pro- and anti-viral effects of polyanionic compounds is still unclear. The interaction between a lower dose of polyanionic compounds with a virus particle might transform its overall structure into one that is more favorable for the engagement of the host receptor. This might lead to more efficient viral entry. This hypothesis needs to be tested in the future to resolve one of the most concerning side effects of CGs.

On the other hand, the formation of amyloid fibrils in semen was also shown to play a positive role in the promotion of HIV infection [78,79]. Since CGs improve the formation of amyloid fibrils, this might be a potential mechanism for the enhancement of HIV infection by CGs [14]. Of note, human defensins 5 and 6, which are produced by cervicovaginal epithelial cells, also significantly enhanced HIV infectivity [68]. They also antagonized the anti-HIV activity of CGs in vitro [68]. Interestingly, higher concentrations of polyanion microbicides, including CGs, suppressed the HIV-enhancing effects of human defensins 5 and 6 [68].

## 4. Herpesviruses

### 4.1. In Vitro Anti-Herpesviruses Activity

Herpesvirus infection is responsible for a wide variety of recurrent diseases such as cold sores, shingles, congenital defects, and several malignancies [80]. More than 90% of the adult population is estimated to be infected with one or more forms of herpesviruses [81]. They include herpes simplex virus type (HSV) 1, Epstein Barr virus (EBV), varicella-zoster virus (VZV), and human cytomegalovirus (HCMV). Fifteen different in vitro studies reported CG antiviral activities against different herpesviruses by using various cell lines such as Vero cells, PPK cells, Hela cells, human foreskin fibroblasts, mouse astrocytes, and MDBK cells [18,21,22,23,24,25,26,31,32,40,43,81,82,83] (Table 6)**.** Quantitation of plaque-forming units and virus-induced cytopathic effects were used to determine their IC_50_ values. They vary from 0.01 to 34.3 µg/mL depending on the cell types, virus types, and experimental systems that were used. Their anti-herpesviruses IC_50_ value range seems to overlap with their anti-HIV IC_50_ value range, which was from 0.03 to 100 µg/mL (Table 3). These data suggest that, similar to HIV, human herpesviruses (e.g., HSV-1, HSV-2, HCMV, and VZV) and animal herpesviruses (e.g., bovine herpesvirus type 1 [BoHV-1] and suid herpesvirus type 1 [SuHV-1]) are susceptible to CG inhibitory activities [18,21,22,23,24,25,26,31,32,40,81,82,83]. In particular, antiherpetic activity was directly correlated with the prevalence of alpha-D-galactose 2,6-disulfate residues in CGs [23]. Like HIV, virus adsorption also seems to be the main target for CG antiviral action since CGs did not affect internalization or early or late protein synthesis of herpesviruses [22]. Of note, anti-herpesvirus activity was increased by partial oxidation of κ-and ι-CGs, which indicates the utility of structurally modifying CGs to improve their antiviral actions against herpesviruses [25]. In line with the key role of sulfated groups in the execution of antiviral activity in other viruses [43,58,59,60], the correlation of CG antiviral activity and the degree of their sulfation was also applicable to herpesviruses. For example, the antiviral IC50 values of fractions obtained from the same algal species against HSV-1 correlated well with their charge density induced by sulfation [23].

### 4.2. In Vivo Anti-Herpesviruses Activity

Fifteen studies examined the antiviral effects of CGs on herpesviruses infection by using mouse and cat models [7,8,40,62,63,82,83,84,85,86,87,88,89] (Table 7). CGs were used either alone or in combination with a variety of other antiviral drugs such as nonoxynol-9, ZA, griffithsin, and MIV-150. Among them, two studies confirmed CG antiviral activity against herpesviruses that did not target viral entry [62,63]. In these two studies, ι-CG augmented NK activity of spleen cells and facilitated the infiltration of polymorphonuclear neutrophils into the peritoneal cavity and inhibited the spread of mouse cytomegalovirus (MCMV) from the peritoneal cavity to the plasma [62]. All of the other 13 studies verified the antiviral activities of CGs against HSV-2 infection via a common antiviral mechanism that involves disruption of viral entry. In particular, the CG-based formulations used by Maguire et al. were significantly more effective against herpesviruses infection than currently marketed spermicides containing the same amount of nonoxynol-9 [90].

The λ-CG 1T1 demonstrated irreversible virucidal action against herpesviruses [84]. By using the cat conjunctiva model, which is caused by feline herpesvirus-1 (FHV-1) infection, the antiviral activities of CGs were further confirmed [88]. In this study, λ type IV CG shortened the duration of the FHV-1-positive period in FHV-1-infected cats [88]. CG, when combined with griffithsin, an algae-derived antiviral lectin protein, synergistically reduced HSV-2 vaginal infection in mice when administered before HSV-2 challenge [86]. Furthermore, this griffithsin/CG combination formulation, when administered in the form of a fast-dissolving insert, also protected mice against HSV-2 infection when applied vaginally [80]. These animal studies clearly demonstrated the superior antiviral activities of CGs in in vivo settings.

### 4.3. CG-Resistant HSV Variants

Due to the constantly mutating nature of RNA viruses, the development of drug resistance is inevitable during long-term usage of antiviral drugs. Therefore, the development of resistance to CGs by susceptible viruses needs to be monitored to assure consistent antiviral efficacy during their application. To gain insight into the development of CG resistance, HSV-1 was grown continuously in the presence of a low dose of CGs for a long period of time. As expected, HSV-1 variants arose after chronic selection with CGs [91]. These CG-resistant HSV-1 variants formed large plaques with an altered syncytial phenotype [91]. However, there was no correlation between the susceptibility of HSV-1 to CGs and its syncytial phenotype [91]. Instead, these CG-resistant HSV-1 variants showed a marked virulence when inoculated intranasally into mice and led to a generalized spread of the virus [92]. Also, mice infected intranasally with two syncytial variants of HSV-1 showed altered expression of cytokines [93]. The resistance-related characteristics of these HSV-1 syncytial variants need to be explored in more detail to understand the underlying mechanism that leads to CG resistance.

## 5. Respiratory Viruses

### 5.1. In Vitro Anti-Respiratory Viruses Activity

In general, respiratory viruses are defined as viruses that cause either upper or lower respiratory tract infections. Typically, they include influenza virus, parainfluenza virus, adenovirus, respiratory syncytial virus (RSV), human rhinovirus (HRV), and coronavirus. Eight studies examined CG in vitro antiviral activities against these respiratory viruses by using different susceptible cell lines (e.g., human nasal epithelial, HeLa, MDCK, and Vero cells) [34,42,94,95,96,97,98,99]. As shown in Table 8, most CGs suppressed respiratory virus propagation with IC_50_ values ranging from 0.04 to 276.5 µM. As with other antiviral studies, CGs blocked the adsorption of respiratory viruses. However, one study reported that the antiviral mechanism of action was independent of viral entry disruption. In this report, CGs inhibited influenza A virus mRNA and protein expression after viral internalization into cells [42]. In addition, ι-CG-containing over the counter (OTC) products such as lozenges and Coldmaris were also highly active against respiratory viruses such as HRV 1a, HRV8, influenza virus A H1N1, coxsackievirus A10, and human coronavirus OC43 [94,97]. Considering the ongoing coronavirus pandemics, a nasal spray of CG-containing OTC products might be helpful for the prevention of coronavirus transmission.

### 5.2. In Vivo Anti-Respiratory Viruses Activity

Five studies tested the in vivo antiviral potency of CGs against respiratory viruses alone or in conjunction with zanamivir, which is a neuraminidase inhibitor of the influenza virus, by using mouse models (Table 9) [96,100,101,102,103]. First, Fujisawa et al. found that type II CGs depleted macrophages and increased polymorphonuclear leukocytes, resulting in an enhanced influenza virus titer [100]. Based on this data, they suggested that type II CG-resistant polymorphonuclear leukocytes play a protective role during the early stages of influenza virus infection [100]. This virus-assisting effect of type II CGs seems to be independent of viral adsorption inhibition [102]. In contrast to this report, three in vivo mouse studies found decreased viral titers, increased host survival, inhibition of pulmonary edema, decreased weight loss, and reduced necropsy and inflammation when mice were pretreated with CGs, and then challenged with influenza A virus [96,98,101]. In particular, infecting mice with a lethal dose of influenza A virus followed by ι-CG treatment protected mice to a similar degree as mice treated with oseltamivir, which is another neuraminidase inhibitor of influenza virus [96]. The intranasal application of zanamivir and κ/ι CG is also synergistically active against influenza A virus in the murine model [98]. One study evaluated the safety of ι-CG via an intranasal route by using animal models [104]. This study revealed no penetration of ι-CG across nasal mucosa and no systemic delivery of ι-CG into the bloodstream [104]. Consistently, no relevant toxic or secondary pharmacological effects due to systemic exposure were observed in the rabbit or rat repeated dose toxicity studies [104]. In addition, no signs of immunogenicity or immunotoxicity and no stimulation of a panel of pro-inflammatory cytokines were observed by the treatment of ι-CG in both in vivo and in vitro models [104].

Five clinical studies also supported the beneficial effects of CGs in alleviating clinical symptoms and reducing viral loads (Table 10) [61,105,106,107,108,109]. In particular, the administration of a ι-CG nasal spray reduced symptoms of the common cold and viral loads in nasal lavages in patients with early symptoms of the common cold [105]. Although ι-CG treatment failed to alleviate acute common cold symptoms in children, it did significantly reduce viral loads in nasal secretions [106]. Another study also reported that direct local administration of CGs via nasal sprays reduced the duration of cold symptoms and viral loads in nasal wash fluids [107].

The low molecular weight version of κ-CG also significantly suppressed mouse-adapted influenza virus-induced pulmonary edema [101]. The administration of a CG nasal spray diminished virus-confirmed common cold duration in children as well as in adults. It also increased viral clearance and reduced symptom relapse [108]. ι-CG also significantly reduced rhinovirus/enterovirus-induced cold symptoms relative to a placebo during the first four days when symptoms were most severe [109]. These data further suggest that CGs have highly active antiviral potentials against a variety of respiratory viruses.

## 6. Human Papillomavirus (HPV)

### 6.1. In Vitro Anti-HPV Activity

High-risk type HPV infection is a major cause of cervical cancer development in women. Five studies examined the in vitro antiviral activities of CGs against different types of HPV including HPV6, 16, 18, 31, 34, 45, and 58 using HeLa, 293T, and NCI-60 cells [20,33,86,110,111] (Table 11). Interestingly, genital HPVs were approximately one thousand-fold more susceptible to CGs since their IC_50_ values against HPVs were all within nanomolar ranges. In addition to this superior antiviral potency, CGs blocked HPV infection through a second, post-attachment heparan sulfate-independent effect in addition to their heparan sulfate-mimicking antiviral mechanism [20]. Since alpha 6 integrin is a major host receptor necessary for HPV infection, CG-induced alpha 6 integrin internalization may also contribute to reduced availability of the host receptor. These three distinct antiviral mechanisms may play a role in facilitating nanomolar CG antiviral activities against HPV infection [86]. Since HPV capsids specifically bind to tumors in a heparan sulfate-dependent manner in vitro and in vivo, ι-CG treatment blocked HPV binding and infection in all tumor lines [110]. In line with this, a CG-based vaginal lubricant maintained in vitro inhibitory activity against HPV infection according to CVLs collected after sexual intercourse [112].

### 6.2. In Vivo Anti-HPV Activity

The in vivo anti-HPV activities of CGs were tested using mouse and macaque models (Table 12). The ι-CG treatment prevented HPV infection even in the presence of nonoxynol-9, which is a vaginal spermicide [113]. This HPV-suppressive property of ι-CG was encouraging since nonoxynol-9 greatly increased HPV infection susceptibility in a previous study [90,113]. A CG-containing gel also caused significantly less HPV infection in a mouse model [33]. A griffithsin-CG combination provided synergistic protection against vaginal HPV infection in mice when they were dosed during and after HPV16 challenge [86]. In particular, a griffithsin-CG combination in the form of a fast-dissolving insert protected mice vaginally against HPV pseudovirus [80]. In clinical studies, the prevalence of HPV infection was lower in compliant CG users than compliant placebo users [114] (Table 13). When the CG gel was substituted for Sortilege during an internal digital examination, a significant decrease in HPV infection was observed [115]. As cytology screening in women induced a transient enhancement of HPV infection susceptibility, CG-based gel administration during examination might help mitigate this potential HPV infection enhancement induced by cytological screening [115]. CG treatment also lowered the number of HPV-infected participants [116]. In addition, CG gel administration accelerated the normal clearance of genital HPV infection in HPV-positive women [117]. All of these data suggest the superior antiviral potency of CGs in both animal and clinical studies.

## 7. Dengue Virus (DENV)

Dengue virus (DENV) causes dengue fever, a mosquito-borne tropical disease. Five different in vitro studies reported CG antiviral activities against DENV using Vero, HepG2, foreskin PH, C6/356 HT mosquito, and BHK-21 cells [16,29,36,38,39] (Table 14). λ-CG inhibitory activities against DENV infection result from dual interference with virus adsorption and internalization of the nucleocapsid into the cytoplasm [36]. More specifically, CG-treated DENV particles were not released from endosomes after entry [36]. However, ι-CG failed to inhibit the adsorption of DENV to C6/36 HT mosquito cells [38]. This failure appeared to be related to the low presence of adequate heparan sulfate in C6/36 HT cell surfaces [38]. The development of CG resistance by DENV was also noticed after serial passaging in Vero cells. However, antiviral susceptibility was not altered in DENV propagated in C6/36 HT mosquito cells [16]. In particular, adsorption kinetics and internalization of resistant DENV variants in Vero cells was significantly diminished, but entry into C6/36 cells was unaffected [37]. A strong inhibitory CG effect was also confirmed by an antiviral assay that monitors cellular oxygen consumption rates [29].

## 8. Other Viruses

Several studies have confirmed the in vitro antiviral activities of CGs against different kinds of viruses including adenovirus [18,118], African swine fever virus [27,35,83,118], arenavirus [17], chikungunya virus [19], coxsackievirus [18], Ebola virus [119], encephalomyocarditis virus [118], enterovirus [120,121], hantavirus [122], hepatitis A virus, measles virus [118], metapneumovirus [123], parainfluenza virus [18], poliovirus [18,118], rabies, reovirus [30], semliki forest virus [19,118], sindbis virus [18], scrapie [124], vaccinia virus [18,118], and vesicular stomatitis virus [18,118] (Table 15). Their IC_50_ values ranged from 0.2 to 400 ug/mL.

## 9. Conclusions and Future Direction

This review examined many of the pharmacological properties of CGs. They include in vitro and in vivo safety profiles; mechanisms of antiviral action; and in vitro and in vivo antiviral activities against HIV, HSV, respiratory viruses, HPV, DENV, and other viruses. Novel phenomena such as enhancement of HIV infectivity and development of CGs-resistant HSV variants found during development processes were also discussed. Despite the rather disappointing outcome of one clinical study, there still seem to be many desirable characteristics of microbicides that use CGs as the main pharmacological ingredient.

Due to the abundance of red algae as a natural resource, its production cost should be relatively low when compared to other chemically synthesized drugs. As a wide distribution of microbicides in the general public is necessary to efficiently prevent transmission of viral infections within society, the low production cost of CG-based microbicides would provide better access to these types of preventive measures.

Due to the broad antiviral spectrum of CGs [1,2,3,16], the use of CG-based microbicides may be the best prophylactic strategy in preventing multiple viral infections simultaneously. The versatile preventive capability of CGs could help suppress many clinically relevant STIs caused by HIV, HSV, and HPV. In addition to their superior antiviral activities against STIs, CGs also demonstrated highly effective inhibitory actions against respiratory viruses such as human rhinovirus, influenza virus, parainfluenza virus, coxsackievirus, and coronavirus, which are responsible for major, clinically-important respiratory infections [96,100,101,102,103]. As shown in Table 10, the clinical application of CG-based nasal sprays produced encouraging results. Considering the ongoing, world-wide COVID-19 pandemic severity, the use of CG-based nasal sprays might help mitigate the spread of coronavirus infections. The development of CG-based sprays as general disinfectants or virucides for public hygiene could be envisaged due to their extreme antiviral coverage.

The thousand-fold increase in their antiviral potency against HPV when compared to HIV and HSV highlights the possibility of developing CG-based microbicides that are specifically designed to prevent HPV infections. The use of CG-based gels could provide an on-demand, pre-exposure prophylactic option to those who are not vaccinated and want to protect themselves against HPV infection.

Numerous examples of successfully combining CGs with other antiviral drugs to synergistically block multiple viral infections emphasize their utility as an antiviral booster. As current anti-HIV drugs are not recommended for single preventive use due to the potential spread of drug resistance, a combination of CGs with anti-HIV drugs, such as AZT and MIV-150, may minimize drug resistance-associated problems.

Despite all of these advantages, there are still many challenges that need to be addressed to successfully develop CG-based microbicides. First, the reason why CG-based gels failed to prevent HIV transmission in clinical trials needs to be explored more thoroughly. Particularly, a more detailed pharmacokinetic study of CGs needs to be performed to exclude the possibility of insufficient CG delivery in target tissues as a contributing factor to the loss of CG-based gel antiviral activity. Also, CG-based gel clinical study designs need to be re-evaluated to ensure they do not affect the quality of the overall conclusions of such a clinical study. Second, HIV infection enhancement by CGs needs to be revisited. This seemingly paradoxical pro-viral action of CGs, specifically at low concentrations, needs to be explored in more detail experimentally and mechanistically. CG dose-dependent differential effects on the overall structures of virus particles and the efficiency of virus entry need to be studied at the molecular level. Neutralizing HIV infectivity enhancement by CGs via chemical alteration should be explored. Third, the molecular mechanism that causes the emergence of CG-resistant viral variants needs to be determined. In particular, genomic analyses of resistant variants should be performed to find potential relationships between specific viral genes and resistance development. The feasibility of delivering high concentrations of CGs to prevent the development of resistance also needs to be tested. Fourth, CG host-targeting antiviral modes of action such as disruption of host receptors and inhibition of intercellular virus transfer need to be studied in more detail. As viruses may be less prone to develop resistance to CG host-dependent antiviral activities, potentiating these CG virus entry-independent antiviral activities by chemical modification should be tested in the future.

To reassess the possibility of a second round of CG pharmacological development as a new class of preventive microbicides, all CG-based microbicide preclinical and clinical development processes were summarized and re-evaluated. Based on the strengths and weaknesses of CGs, the direction of a second CG development round was proposed. This bird’s-eye view of the various pharmacological characteristics of CGs will help provide future research directions for the successful development of CG-based antimicrobial prophylactics.

## Figures and Tables

**Table 1:** In vitro CG toxicities. (CC_50_, half-maximal cytotoxicity concentration; MDCK cells, Madin–Darby canine kidney cells; MDBK cells, Madin–Darby bovine kidney cells; BHK-21 cells, baby hamster kidney 21-cells). When more than two CGs co-exist as a hybrid, they were listed together with a dash.

CG Type	Cell Line	CC_50_ (µg/mL)	Ref
κ-CG	MDCK cells	>250	[34]
κ-CG	Vero cells	>1000	[43]
κ-CG	PRK cells	>100	[18]
κ-CG	Vero cells	2000	[27]
κ-CG CO-1	MDCK and A549 cells	857	[42]
ι-CG	Vero cells	250	[35]
ι-CG	Vero cells	>1000	[21]
ι-CG	Vero cells	>500	[19]
ι-CG	Vero and C6/36 HT mosquito cells	>1000	[16]
ι-CG	Vero cells	>400	[32]
ι-CG	Vero cells	>5000	[43]
λ-CG	Vero cells	400	[17]
λ-CG	Vero cells	>10	[31]
λ-CG	Vero cells	>1000	[23]
λ-CG	MDBK cells	>1000	[26]
λ-CG	Vero and C6/36 HT mosquito cells	>1000	[37]
λ-CG	BSR cells	>1000	[30]
λ-CG	MT-4 cells	300	[41]
λ-CG	Vero cells	>5000	[43]
λ-CG	PRK cells	>100	[18]
λ-CG	Vero cells	3000	[27]
κ/λ-CG (PC-515)	Hela cells	>16	[33]
Oxidized κ/ι-CG	Vero cells	>1000	[25]
ι/λ-CG	HeLa cells	>100	[20]
κ/ι/λ-CG	PLC/PRF/5 cells	>1000	[28]
κ/ι/λ-CG	Vero, HepG2 and foreskin PH cells	>1000	[36]
κ/ι/λ-CG	Vero and C6/36 HT mosquito cells	>1000	[38]
κ/ι-CG, λ-CG, and υ/ν-CG	Vero and human foreskin fibroblast cells	>1000	[22]
κ/ι-CG, λ-CG, and υ/ν-CG	Mouse astrocytes and Vero cells	>1000	[24]
κ/ι/ν-CG	Vero, HepG2, and foreskin cells	>1000	[39]
κ/ι/ν-CG C2	Vero cells	>1000	[40]
Unspecified CG	BHK-21 fibroblast cells	>5	[29]

**Table 2:** CG in vivo safety in clinical studies. (STI, sexually transmitted infections; CVL, cervicovaginal lavage; HIV(−), HIV-negative; HIV(+), HIV-positive; MIV-150, nonnucleoside reverse transcriptase inhibitor; ZA, zinc acetate).

CG Type	Human Subject	Duration and Frequency	Effects	Ref
2% ι-CG (PC-213)	25 women	Once a day for 1 week	No irritation of the female reproductive tract by colposcopy	[44]
3% κ/λ-CG (PC-515)	165 women	4 times per week for 1 year	No abnormal clinical signs or symptoms, no vaginal flora change, and highly acceptable	[51]
3% κ/λ-CG (PC-515)	55 HIV(−) couples	Before sexual intercourse for 6 months	Highly acceptable and increased sexual pleasure	[48]
3% κ/λ-CG (PC-515)	60 HIV(+) healthy women and men	Once a day or before sexual intercourse for 2 weeks	Highly acceptable	[52]
3% κ/λ-CG (PC-515)	60 HIV(+) healthy women and men	Once a day or before sexual intercourse for 2 weeks	No abnormal clinical signs or symptoms, no vaginal flora change, and no genital shedding of HIV RNA	[46]
3% κ/λ-CG (PC-515)	55 HIV(−) women	Before sexual intercourse for 1 month	Intact epithelium and no increased proinflammatory cytokines in CVL	[47]
3% κ/λ-CG (PC-515)	55 HIV(−) couples	2–3 times per week before sexual intercourse for 6 months	Generally acceptable, no epithelial disruption, and no abnormal genital flora	[45]
3% κ/λ-CG (PC-515)	60 HIV(+) women crossover	Once daily for 1 week followed by 3-week wash-out for 1 month	Reduced HIV viral load in CVL and well tolerated	[50]
3% κ/λ-CG (PC-515)	400 HIV(−) women	3 times a week before sexual intercourse for 6 months	No genital irritation or epithelial disruption, no difference in STI rate, and generally acceptable	[53]
3% κ/λ-CG (PC-515)	60 HIV(+) women crossover	Once-daily for 1 week for 3 months with cross-over	Highly acceptable	[54]
3% κ/λ-CG + MIV-150 + ZA (PC-1005)	25 HIV(−) women	Once-daily for 2 weeks	Well tolerated and maintenance of anti-HIV and anti-HPV activity in CVL	[49]

**Table 3:** CG in vitro anti-HIV activities. (AZT, azido-3′-deoxythymidine; ELISA, enzyme-linked immunosorbent assay).

CG Type	Experimental System	Antiviral Assay	IC_50_ (µg/mL)	Ref
λ-CG	MT-4 cells infected with HIV-HTLVIIIB	Expression of HIV-specific antigens and plaque reduction assays	9500 (IU/mL)	[61]
ι-CG	MT-4 cells infected with HIV	ELISA of p24 antigen	100	[56]
ι-CG and κ-CG	MT-4 cells and ME-180 cells infected with HIV	ELISA of p24 antigen	1.6 and 4	[55]
Depolymerized λ-CG	MT-4 cells infected with HIV-HTLVIIIB	Expression of HIV-specific antigens	3.9 (IC_100_)	[66]
κ-CG and λ-CG	MT-4 cells infected with HIV-HTLVIIIB	Expression of HIV-specific antigens	12 and 1.9	[18]
κ-CG and AZT conjugates	MT-4 cells infected with HIV (BRU)	Quantitation of virus-induced cytopathicity	0.1	[41]
λ/κ-CG + MIV-150	CEM-SS cells infected with HIVMN and HIV-2CDC310342	Microtiter syncytial assay	0.1–13.8	[67]
λ/κ-CG (PC-515)	TMZ.bI and DC cells infected with HIV-Bal	Quantitation of β-gal and ELISA of p24 antigen	0.03–4.17, 1.61	[12]
ι-CG	HeLa-CD4-CCR5 cells infected with HIVJR-FL	Luciferase assay	1–10	[68]
λ-CG	P4-R5 MAGI cells	ELISA of p24 antigen	3.7 and 64	[13]
λ-CG	MT-2 cells infected with HIV BaL and IIIB	ELISA of p24 antigen	0.58 and 0.68	[14]

**Table 4:** CG ex vivo anti-HIV activities. (MIV-150, nonnucleoside reverse transcriptase inhibitor; ZA, zinc acetate; RT-SHIV, Simian immunodeficiency virus mac239 bearing HIV reverse transcriptase).

CG Type	Experimental Systems	Antiviral Assay	Effects	Ref
λ/κ-CG (PC-515)	Human cervical explants infected with HIVBaL	ELISA of p24 antigen	50% inhibition of infection	[71]
λ/κ-CG + MIV-150 + ZA (PC-1005)	Human cervical explants incubated with CVLs and then infected with HIVBaL	ELISA of p24 antigen	Inhibition of infection	[9]
λ/κ-CG + MIV-150 + ZA (PC-1005)	Macaque vaginal explants infected with SHIV-RT	ELISA of p24 antigen	Inhibition of infection	[7]
λ/κ-CG + MIV-150 + ZA (PC-1005)	Macaques vaginal explants challenged with SHIV-RT	Quantitation of viral DNA and ELISA of SIVmac p27	Inhibition of infection	[69]
λ/κ-CG + MIV-150 + ZA (PC-1005)	Macaques vaginal explants challenged with SHIV-RT infected PBMCs	Quantitation of viral DNA and ELISA of SIVmac p27	Inhibition of infection	[70]

**Table 5:** CG in vivo anti-HIV activities. (MIV-150, nonnucleoside reverse transcriptase inhibitor; PBMC, peripheral blood mononuclear cells; ELISA, enzyme-linked immunosorbent assay; SHIV-RT, Simian immunodeficiency virus mac239 bearing HIV reverse transcriptase, TFV; tenofovir).

CG Type	Efficacy Model	Dose	ADMINISTRATION	Antiviral Assay	Effects	Ref
λ/κ-CG	Inoculation of stained mouse macrophages into the vagina of mice	20 µL of stock	Single inoculation	Counting the number of macrophages in lymph nodes	90% inhibition	[72]
λ/κ-CG + TFV + Zn + MIV-150	Macaques vaginally challenged with SHIV-RT	3 mL of 3% gel	Single vaginal	Plasma viral load quantitation	Inhibition of vaginal transmission and no difference in antiviral activity	[7]
λ/κ-CG + MIV-150 (PC-817)	Macaques rectally challenged with SHIV-RT	3 mL of 3% gel	Single rectal	Plasma viral load quantitation	Inhibition of rectal transmission	[12]
λ/κ-CG + MIV-150 + ZA (PC-1005)	Macaques either vaginally or rectally challenged with SHIV-RT	2 mL of 3% gel	Single vaginal	Plasma viral load quantitation	Inhibition of infection	[74]
λ/κ-CG + MIV-150 + ZA (PC-1005)	Macaques either vaginally or rectally challenged with SHIV-RT	2 mL of 3% gel	Single vaginal or rectal	Plasma viral load quantitation	Complete and limited inhibition of vaginal or rectal transmission	[73]
λ/κ-CG + ZA	Macaques either vaginally or rectally challenged with SHIV-RT	2 mL of 3% gel	Vaginal for 2 weeks	Plasma viral load quantitation	Inhibition of infection	[75]
λ/κ-CG + MIV-150 + ZA (PC-1005)	Macaques vaginal explant challenged with either free or cell-associated SHIV-RT	1:100 and 1:300 dilution	Immersion of explant with diluted gels	Quantitation of viral DNA and ELISA of SIVmac p27	Inhibition of infection	[8]
λ/κ-CG	Preventive effects on 6202 HIV(−) women	4 mL of 3% gel	Before sexual intercourse for 9–24 months	HIV incidence by seroconversion	No difference in HIV incidence	[11]

**Table 6:** CG in vitro anti-herpesvirus activities. (HSV, herpes simplex virus; CMV, cytomegalovirus; VZV, varicella-zoster virus; PFU, plaque-forming unit; CPE, cytopathic effect; PPK cells, primary porcine kidney cells; BoHV-1, bovine herpesvirus type 1; SuHV-1, suid herpesvirus type 1; MDBK cells, Madin–Darby bovine kidney cells).

CG Type	Herpes Virus Type	Cell Line	Antiviral Assay	IC_50_ (µg/mL)	Ref
ι-CG	HSV-1 and 2	Vero cells	PFU	2 and 10	[83]
λ/κ-CG	HSV-1 and 2	PPK cells	CPE	3.7, 1.6, 2, 1.5	[18]
λ/κ-CG	CMV	Hela cells	CPE	2.8/0.3	[18]
λ-CG	HSV-1 and 2	Vero cells	CPE and PFU	<100	[31]
λ-CG	HSV-1 and 2	Vero cells	CPE and PFU	0.3	[23]
κ-CG	HSV-2	Human foreskin fibroblast cells	CPE	0.01	[81]
λ-CG type IV, ι-CG type V	HSV-2	HeLa cells	CPE	2.4 and 1.4	[82]
λ-CG 1T1, κ/ι-CG 1C1, µ/ν-CG 1C3	HSV-1 strain F and HSV-2 strain G	Vero cells and human diploid foreskin fibroblast cell line PH	PFU	0.4–3.3	[22]
λ-CG 1T1, κ/ι-CG 1C1, µ/ν-CG 1C3	HSV-1 strain F and HSV-2 strain G	Mouse astrocytes and Vero cells	PFU	0.4–3.6	[24]
ι-CG	HSV-1 and 2	Vero cells	PFU	0.65–9.33	[21]
κ/ι/ν-CG, C2	HSV-1 strain F and HSV-2 strain G	Vero cells	PFU and survival	0.5–5.6	[40]
Oxidized k- and ι-CG	HSV-1 and 2	Vero cells	PFU	0.98–34.3	[25]
λ-CG	BoHV-1 and SuHV-1	MDBK cells	PFU	0.52 and 10.42	[26]
ι-CG	HSV-1	Vero cells	Neutral red dye	6.31	[32]
κ/ι/λ-CG	VZV	Vero cells	PFU	0.5/0.8/1.8	[43]

**Table 7:** CG in vivo anti-herpesvirus activities. (PFU, plaque-forming unit; LD_50_, half-maximal lethal dose; MCMV, murine cytomegalovirus; FHV-1, feline herpesvirus-1; CPE, cytopathic effect; FDI, fast-dissolving insert; qPCR, quantitative polymerase chain reaction).

CG Type	Efficacy Model	Dose	Antiviral Assay	Administration	Effects	Ref
ι-CG type V	ICR mice infected with MCMV	0.5 mg	PFU	Intraperitoneal	Decreased mortality and titer and increased PFU/LD_50_	[63]
ι-CG type V	ICR mice infected with MCMV	0.5 mg	PFU	Intraperitoneal	Inhibition of viral spread from the peritoneal cavity to the plasma	[62]
λ/κ/ι-CG	BALB/c mice infected with HSV-2	0.05–1%	PFU in vaginal secretion	Vaginal	Inhibition of infection	[89]
κ-CG	C57B1/6 mice infected with HSV-2	0.1–100 mg/mL	Shedding of virus, visible lesions, and CPE	Vaginal	Inhibition of infection	[81]
CG-based nonoxynol-9	BALB/c mice infected with HSV-2	20 ul of 3%	Symptom of infection	Vaginal	Inhibition of infection	[90]
λ-CG type IV (1T1), ι-CG type V	Swiss Webster mice infected with HSV-2	10 mg/mL	Sign of disease	Vaginal	Inhibition of infection	[82]
λ-CG	BALB/c mice infected with HSV-2	2 and 3%	Survival	Vaginal	100% survival	[87]
λ-CG 1T1	BALB/c mice infected with HSV-2	10 mg/mL	PFU and survival	Vaginal	90% survival and no virus shedding	[84]
κι/ν-CG C2	BALB/c mice infected with HSV-2	8 mg/mL	PFU and survival	Vaginal	70% survival	[40]
λ-CG type IV	FHV-1 induced conjunctivitis in cats	250 µg/mL	PFU	Topical	Reduction of virus titers but no alteration in the clinical course	[88]
λ/κ-CG + ZA	BALB/c mice infected with HSV-2	20 µL of 3%	Survival	Vaginal and rectal	Inhibition of infection	[85]
λ/κ-CG + ZA	BALB/c mice infected with HSV-2	20 µL of 3%	Survival	Vaginal and rectal	Inhibition of infection	[8]
CG + griffithsin	BALB/c mice infected with HSV-2	50 µL of griffithsin solution (19.1 mg/mL)	Infection rate	Vaginal	Inhibition of infection	[86]
CG + MIV-150 + ZA	BALB/c mice infected with HSV-2	10 µL of stock	qPCR	Vaginal	Inhibition of infection	[7]
CG + griffithsin	BALB/c mice infected with HSV-2	10 µL of 0.1% stock	qPCR	Vaginal	Inhibition of infection	[80]

**Table 8:** CG in vitro anti-respiratory virus activities. (TCID_50_, median tissue culture infectious dose; MDCK cells, Madin–Darby canine kidney cells; PFU, plaque-forming unit; CPE, cytopathic effect; IF, immunofluorescence; HRV, human rhinovirus).

CG Type	Virus Type	Cell Line	Anti-Viral Assay	IC_50_ (µg/mL)	Ref
ι-CG	HRV 1A, 2, 8, 14, 16, 83, and 84	Human nasal epithelial and HeLa cells	TCID_50_	5–10	[95]
ι-CG	Influenza virus strain H1N1 (A/PR8/34) and the formerly pandemic H3N2 (A/Aichi/2/68)	MDCK cells and human nasal epithelial cells	PFU	0.04–0.2	[96]
κ-CG	Influenza A virus	MDCK and A549 cells	TCID_50_ assay and RT-PCR	32.1	[42]
ι/κ/ν-CG hybrid	Influenza A H1N1 virus	MDCK cells	CPE inhibition	276.5	[99]
ι-CG + zanamivir	Influenza A virus H1N1(09)pdm, H3N2, H5N1, and H7N7	MDCK cells	Immunostaining	0.39–11.8	[98]
κ-CG	Swine pandemic influenza A virus H1N1	MDCK cells	TCID_50_, CPE experiments, IF, and Western blot	89.57	[34]
ι-CG (lozenges)	HRV 1a, HRV8, influenza virus A H1N1, coxsackievirus A10, and human coronavirus OC43	HeLa, MDCK, and Vero cells	TCID_50_ and PFU, agglutination assay	234–4524 dilution	[97]
ι-CG and xylometazoline hydrochloride (Coldamaris)	HRV 1a, HRV8, and human coronavirus OC43	HeLa, MDCK, and Vero cells	TCID_50_ and PFU, agglutination assay	16.5, 1.66, and 0.024	[94]

**Table 9:** CG anti-respiratory virus activities in vivo animal studies. (MDCK cells, Madin–Darby canine kidney cells; PMN, polymorphonuclear leukocytes; BID, twice a day; RSV, respiratory syncytial virus).

CG Type	Virus Type	Experimental Model	Dose	Antiviral Assay	Effects	Ref
Type II CG	Influenza virus H1N1 (A/PR/8/34)	BALB/c mice intranasal infection	1 mg/mL	Virus titration with MDCK cells	Depleted macrophages and increased PMN in the blood, enhancement of viral titer	[100]
Type II CG	Influenza virus H1N1 (A/PR/8/34)	BALB/c mice intranasal infection	200 mg/kg	Virus titration with MDCK cells	Depleted macrophages, phagocytes, and monocytes	[102]
ι-CG	Influenza virus strain H1N1 (A/PR8/34) and the formerly pandemic H3N2 (A/Aichi/2/68)	C57Bl/6 mice intranasal infection	60 µg BID for 15 days	PFU	Reduced viral titers and increased survival (40%)	[96]
κ-CG (low MW)	Mouse adapted influenza virus A/FM/1/47(H1N1)	ICR mice with nasal drip	40 µL of 1.5 mg/mL for 7 days	Measure pulmonary edema index	Inhibition of pulmonary edema in mice	[101]
κ/ι-CG and zanamivir	Influenza A virus H1N1(09)pdm, H3N2, H5N1, and H7N7	C57BL/6 mice with intranasal injection	50 µL of 1.2 and 0.4 mg/mL stock BID for 5 days	Survival and weight loss, necropsy, and inflammation	Increased survival, decreased weight loss, reduced necropsy, and inflammation	[98]

**Table 10:** CG anti-respiratory virus activities in clinical studies. (BID, twice a day; RSV, respiratory syncytial virus).

CG Type	Virus Type	Experimental Model	Dose	Antiviral Assay	Effects	Ref
ι-CG (Coldmaris)	Respiratory viruses (influenza, parainfluenza, coronavirus, rhinoviruses, and human metapneumovirus)	35 human subjects, nasal spray, symptom scores	0.12%	Measure viral loads in nasal lavages	Lowered symptom scores and viral loads, lowered proinflammatory cytokines	[105]
ι-CG (Coldmaris)	Respiratory viruses (influenza, parainfluenza, coronavirus, RSV, rhinoviruses, and human metapneumovirus)	213 young human subjects, nasal spray, symptom scores	0.12%	Measure viral loads in nasal lavages	Lowered viral loads but no effects on symptom scores	[106]
ι-CG (Coldmaris)	Respiratory viruses (influenza, parainfluenza, coronavirus, RSV, rhinoviruses, and human metapneumovirus)	211 patients intranasal spray	0.12%	Measure viral loads in nasal lavages	Reduced duration of disease, alleviation of symptom, and reduced viral titers	[107]
ι-CG	Rhinovirus, coronavirus, and influenza A virus	254 human subjects with nasal spray	0.12% TID for 7 days	Nasal lavage sample	Reduced duration of disease, increased viral clearance, and reduced relapses of symptom	[108]
ι-CG	Rhinovirus, coronavirus, and influenza A virus	200 human subjects with nasal spray	0.12% QID for 4–10 days	Nasal lavage sample	No difference in total symptom scores and more effective in coronavirus	[109]

**Table 11:** CG in vitro anti-HPV activities. (GFP, green fluorescence; RFP, red fluorescence; FACS, Fluorescence-activated cell sorting; PsVs, pseudoviruses).

CG Type	Virus Type	Experimental System	Anti-Viral Assay	IC_50_ (ng/mL)	Ref
λ/ι-CG	HPV16	HeLa cells infected with pseudovirus with GFP	GFP assay	5–44	[20]
κ/λ-CG (PC-515)	HPV16, 18, and 45 PsVs	HeLa cells infected with pseudovirus with luciferase	Luciferase assay	1–20	[33]
CG	HPV16, 18, 31, 34, 58, and 6 with luciferase reporter	293T cell infected with furin-cleaved HPV16 pseudovirus	Luciferase assay	250–1000	[111]
CG	HPV16	HeLa cells infected with pseudovirus with luciferase	Luciferase assay	38.6	[86]
1% ι-CG	HPV 16 VLP and PsV	NCI-60 cells infected with RFP-encoded HPV pseudovirus	GFP assay by FACS	N/D	[110]

**Table 12:** CG’s in vivo anti-HPV activities in animal studies. (GFP, green fluorescence protein; RFP, red fluorescence protein; FDI, fast-dissolving insert; CVL, cervicovaginal lavage).

CG Type	Virus Type	Experimental System	Anti-Viral Assay Readout	Effects	Ref
ι-CG (1%)	HPV16	Mouse cervicovaginal mucosa infected with RFP-encoded pseudovirus with GFP capsid	GFP and RFP assay	Inhibition of infection	[113]
ι-CG (1%)	HPV16	12 rhesus macaques infected with RFP-encoded pseudovirus with GFP capsid	Immunohistochemistry of the infected cervix tissue	Decreased infection that was enhanced by cytologic examination	[115]
3% λ/κ-CG (PC-515) + divine 9	HPV16	Mouse cervicovaginal mucosa infected with RFP-encoded pseudovirus with GFP capsid	GFP and RFP assay	Inhibition not affected by seminal plasma	[33]
Griffithsin + CG	HPV16	Mouse cervicovaginal mucosa infected with luciferase-encoded pseudovirus	Luciferase	Inhibition	[86]
CG-based lubricant (Divine 9)	HPV16	Antiviral activity of cervicovaginal lavage (CVL) using 293T cells infected with SEAP-encoded PsV	RFP assay	Inhibition	[112]
Griffithsin + CG	HPV	Balb/C mice equipped with FDI infected with luciferase-encoded HPV16 pseudoviruses	Luciferase	Inhibition	[80]

**Table 13:** CG’s anti-HPV activities in clinical studies. (GFP, green fluorescence protein; RFP, red fluorescence protein; FDI, fast-dissolving insert; CVL, cervicovaginal lavage).

CG Type	Virus Type	Experimental System	Anti-Viral Assay Readout	Effects	Ref
λ/κ-CG (carraguard)	High-risk HPVs	1718 women used gel plus condoms during each act of vaginal intercourse	Prevalence of high-risk HPV infection by Pap smear	Lower prevalence of high-risk HPV infection	[114]
CG-based lubricant (Divine 9)	HPV	280 women used gel plus condoms during each act of vaginal intercourse	Prevalence of high-risk HPV infection by Pap smear	Lower prevalence of HPV infection	[116]
0.02% CG and Propionibacterium extract (CGP)	HPV	40 HPV-infected women	Clearance of HPV infection	Accelerated the clearance of HPV infection (26 to 60% clearance rate)	[117]

**Table 14:** CG in vitro anti-DENV activities. (BHK-21 cells, baby hamster kidney 21 cells).

CG Type	Virus Type	Experimental System	Anti-Viral Asssay	IC_50_ (µg/mL)	Ref
κ/ι/ν-CG	DENV2	Vero cells, human hepatoma HepG2, and foreskin PH cells	Plaque formation	1	[39]
ι/λ/κ-CG	DENV1, 2, 3, 4	Vero cells, human hepatoma HepG2, and foreskin PH cells	Plaque formation	0.1–40.7	[36]
ι/λ/κ-CG	DENV2	Vero cells and C6/36 HT mosquito cells	Plaque formation	0.4/7	[38]
ι-CG	DENV2	Vero cells and C6/36 HT mosquito cells	Plaque formation	22.5/0.64	[16]
CG	DENV2	BHK-21 fibroblast cells	Measuring cellular oxygen consumption rate	10	[29]
λ-CG	DENV2	Vero cells and C6/36 HT mosquito cells	Plaque formation	>50	[37]

**Table 15:** CG in vitro antiviral activities on other viruses. (N/D, not determined).

Virus Type	CG Type	Cell Line	IC_50_ or CPE_50_ (µg/mL)	Ref
Adenovirus type 2	κ and λ	Hela cells	>200/>200	[18]
Adenovirus type 5	ι	Hela cells	>200	[118]
African swine fever virus	ι	Vero cell	10	[118]
African swine fever virus	ι	Vero cell	50	[35]
African swine fever virus	κ and λ	Vero cell	25/150	[27]
Arenavirus	λ	Vero cells	0.2–0.3	[17]
Chikungunya virus	ι	Vero cells	3.8	[19]
Cosackiesvirus type B4	κ and λ	Hela cells	>400/>400	[18]
Ebola virus	ι	Caco-2 cells	N/D	[119]
Encephalomyocarditis virus	ι	Not specified	10	[118]
Enterovirus 71	κ	Vero cells	10–100	[120]
Enterovirus 71	ι	Vero cells	17.8	[121]
Hantavirus	ι	Vero cells and mouse macrophages		[122]
Hepatitis A virus	κ, ι and λ	PLC/PRF/5 cells	2.5, 4.5, and 100.3	[28]
Measles	ι	Not specified	>200	[118]
Metapneumoniavirus	ι	Vero and human bronchial epithelial cells (BEAS-2B)	0.1–1	[123]
Parainfluenza virus type 3	κ and λ	Vero cells	>40/>4	[18]
Polio type 1	ι	Hela cells	>200	[118]
Polio type 1	κ and λ	Hela cells	>400/>400	[18]
Rabies	λ P32	BSR cells	15–57	[30]
Reovirus type 1	κ and λ	Vero cells	>40/>4	[18]
Semliki Forest virus	ι	Vero cells	0.7	[19]
Semliki Forest virus	ι	BHK-21 cells	10	[118]
Sindbis virus	κ and λ	Vero cells	7/2	[18]
Scrapie	λ type IV	Female compton mouse infected with scrapie	inhibition of infection	[124]
Vaccinia	ι	PPK cells	10	[118]
Vaccinia	κ and λ	PPK cells	36/16	[18]
Vesicular stomatitis virus	ι	PRK cells	>200	[118]
Vesicular stomatitis virus	κ and λ	PRK cells	0.3/0.2	[18]
Vesicular stomatitis virus	κ and λ	Hela cells	7/4	[18]
